# Public health and social measures to mitigate the health and economic impact of the COVID-19 pandemic in Turkey, Egypt, Ukraine, Kazakhstan, and Poland during 2020–2021: situational analysis

**DOI:** 10.1186/s12889-022-13411-6

**Published:** 2022-05-17

**Authors:** Noriko Kitamura, Kaja Abbas, Dilip Nathwani

**Affiliations:** 1 Department of Infectious Disease Epidemiology, London School of Hygiene & Tropical Medicine, Keppel Street, London, WC1E 7HT UK; 2 School of Tropical Medicine and Global Health, Nagasaki University, Nagasaki, Japan; 3 Ninewells Hospital and Medical School, University of Dundee, Dundee, DD91SY Scotland UK

**Keywords:** COVID-19, Outbreak response, Vaccine, Infectious disease, Health system, Sustainable development goals

## Abstract

**Background:**

The COVID-19 pandemic had a colossal impact on human society globally. There were similarities and differences in the public health and social measures taken by countries, and comparative analysis facilitates cross-country learning of contextual practices and sharing lessons to mitigate the COVID-19 pandemic impact. Our aim is to conduct a situational analysis of the public health and social measures to mitigate the health and economic impact of the COVID-19 pandemic in Turkey, Egypt, Ukraine, Kazakhstan, and Poland during 2020–2021.

**Methods:**

We conducted a situational analysis of the COVID-19 pandemic response in Turkey, Egypt, Ukraine, Kazakhstan, and Poland from the perspectives of the health system and health finance, national coordination, surveillance, testing capacity, health infrastructure, healthcare workforce, medical supply, physical distancing and non-pharmaceutical interventions, health communication, impact on non-COVID-19 health services, impact on the economy, education, gender and civil liberties, and COVID-19 vaccination.

**Results:**

Since the onset of the COVID-19 pandemic, Turkey, Egypt, Ukraine, Kazakhstan, and Poland have expanded COVID-19 testing and treatment capacity over time. However, they faced a shortage of healthcare workforce and medical supplies. They took population-based quarantine measures rather than individual-based isolation measures, which significantly burdened their economies and disrupted education. The unemployment rate increased, and economic growth stagnated. Economic stimulus policy was accompanied by high inflation. Despite the effort to sustain essential health services, healthcare access declined. Schools were closed for 5–11 months. Gender inequality was aggravated in Turkey and Ukraine, and an issue was raised for balancing public health measures and civil liberties in Egypt and Poland. Digital technologies played an important role in maintaining routine healthcare, education, and public health communication.

**Conclusions:**

The COVID-19 pandemic has exposed weaknesses in healthcare systems in the emerging economies of Turkey, Egypt, Ukraine, Kazakhstan, and Poland, and highlighted the intricate link between health and economy. Individual-level testing, isolation, and contact tracing are effective public health interventions in mitigating the health and economic impact of the COVID-19 pandemic in comparison to population-level measures of lockdowns. Smart investments in public health, including digital health and linking health security with sustainable development, are key for economic gain, social stability, and more equitable and sustainable development.

## Introduction

COVID-19 is the largest pandemic in more than 100 years with its impact felt at national, regional, and global levels. Many countries faced challenges with shortages of resources and capacity in the health sector during their response against the pandemic. A view of public health objectives and economic gains as a trade-off tended to prevent rapid and cross-sectoral response in some countries.

Pandemic-related restrictions disrupted global supply chains, inhibited investment, and interrupted labour markets, affecting the livelihoods of millions of people. The public health crisis has had dire economic consequences on a global scale. The cumulative financial costs of the COVID-19 pandemic in the United States alone by the end of 2021 were estimated at more than US$ 16 trillion, which is 90% of the Gross Domestic Product (GDP) of the US [1].

Progress towards the Sustainable Development Goals (SDGs) attained before COVID-19 has reverted since the onset of the COVID-19 pandemic. The World Bank has estimated that poverty rates have increased for the first time in the last 20 years due to the pandemic as, globally, 90 million people fell into extreme poverty [2]. Education was highly disrupted; the United Nations Educational, Scientific and Cultural Organization (UNESCO) predicted that 100 million children would fall below the minimum reading proficiency level [3]. Child marriage and gender-based violence consequently increased [4, 5]. Routine health services were compromised, and childhood vaccination programmes have halted in 70 countries during the pandemic [6].

The spread of misinformation during epidemics has been documented before, but COVID-19 has brought with it a global deluge of misinformation [7]. The politicisation of the pandemic in many countries led to some politicians being a leading source of misinformation, while an initial underestimation of the pandemic by key public health stakeholders led to inconsistent messaging and public confusion.

A successful rollout of the COVID-19 vaccine is a key for mitigating the health impact of the pandemic as well as boosting confidence in economic activities. Vaccination against COVID-19, prioritising healthcare workers and the elderly, started in many countries in December 2020. However, vaccine supply constraints and vaccine hesitancy in the community pose critical challenges in scaling up coverage and limiting COVID-19 morbidity and mortality [8].

There are similarities and differences in the public health and social measures taken by countries. Comparative analysis facilitates cross-country learning of contextual practices and shares lessons to mitigate the COVID-19 pandemic impact. While COVID-19 hit western Europe hard, little attention has been paid to Western Asia, Northeast Africa, Eastern Europe [9].Central Asia, and Central Europe. To address this evidence gap, the European Bank for Reconstruction and Development commissioned this study to generate country-specific evidence for Turkey (Western Asia), Egypt (Northeast Africa), Ukraine (Eastern Europe). Kazakhstan (Central Asia), and Poland (Central Europe). Therefore, we conducted a situational analysis of the public health and social measures to mitigate the health and economic impact of the COVID-19 pandemic in Turkey, Egypt, Ukraine, Kazakhstan, and Poland during 2020–2021.

## Methods

### Situational analysis

**Fig. 1:**
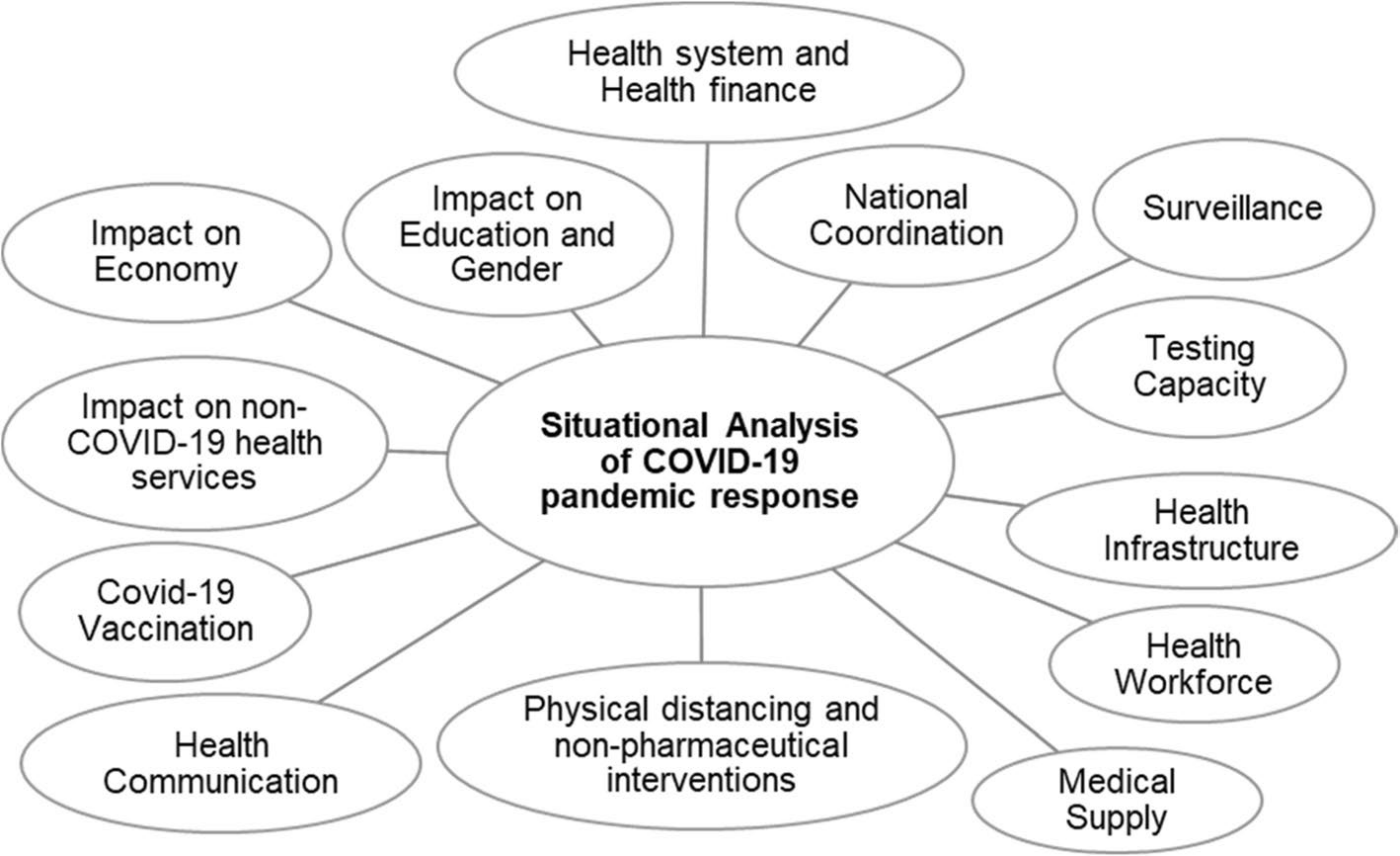
Situational analysis framework of COVID-19 pandemic response and impact. Situational analysis framework of the public health and social measures to mitigate the health and economic impact of the COVID-19 pandemic in Turkey, Egypt, Ukraine, Kazakhstan, and Poland during 2020–2021

We compared the COVID-19 pandemic response in Turkey, Egypt, Ukraine, Kazakhstan, and Poland during 2020–2021 from the perspectives of the health system and health finance, national coordination, surveillance and testing capacity, health infrastructure, healthcare workforce, medical supply, physical distancing and non-pharmaceutical interventions, health communication, impact on non-COVID health services, impact on the economy, education, gender, and civil liberties, and COVID-19 vaccination (see Fig. 1).

We obtained information about each country’s health system and health finance to evaluate pre-pandemic conditions in the five countries. For assessing the appropriateness of the response activities in each country, we collected information about national-level coordination, surveillance capacity, including testing, health infrastructure and workforce, medical supply system, physical distancing measures, and communication strategy. We also assessed the pandemic impact on non-COVID-19 health services, economy, education, and gender in each country.

### Data sources

Each country’s data were collected from publicly available data sources. Academic literature related to response activities to COVID-19 and its socio-economic impact in five countries were searched on EMBASE and PubMed. Each country’s Ministry of Health (MoH) websites, websites of various international organizations, such as World Health Organization (WHO), United Nations Children’s Fund (UNICEF), United Nations Population Fund (UNFPA), the World Bank, United Nations Development Programme (UNDP), United Nations High Commissioner for Refugees (UNHCR), and United Nations Entity for Gender Equality and the Empowerment of Women (UN Women), and web-based news or magazines were also searched. Auto English translation function on the web browser was applied for non-English websites when necessary, and we interpreted the translated material with caution to minimise any misinterpretation or loss in translation.

We collected data on the number of COVID-19 cases, deaths, and vaccination coverage from WHO dashboard [10] and “Our World in Data” [11] database as of 16 November 2021. “WHO Regional Office for Europe” [12] were searched for obtaining information about health systems and health finance in each country. The websites of “COVID-19 health response monitor” [13] and “Health system response monitor” [14] were searched to collect the response activities. Health and economic indicators and demography data were extracted from the World Bank database [15]. The number of hospital beds and physicians per 1,000 people, health expenditure, GDP per capita, and poverty rate were also extracted from the World Bank data base [16] for the baseline resource for health before the onset of the COVID-19 pandemic. The duration of school closures in each country was obtained from United Nations Educational Cultural Organization (UNESCO) website as of 16 November 2021 [3]. Economic impact of COVID-19 in each country was mainly searched in the World Bank’s report or country information websites [17].

## Results

**Fig. 2:**
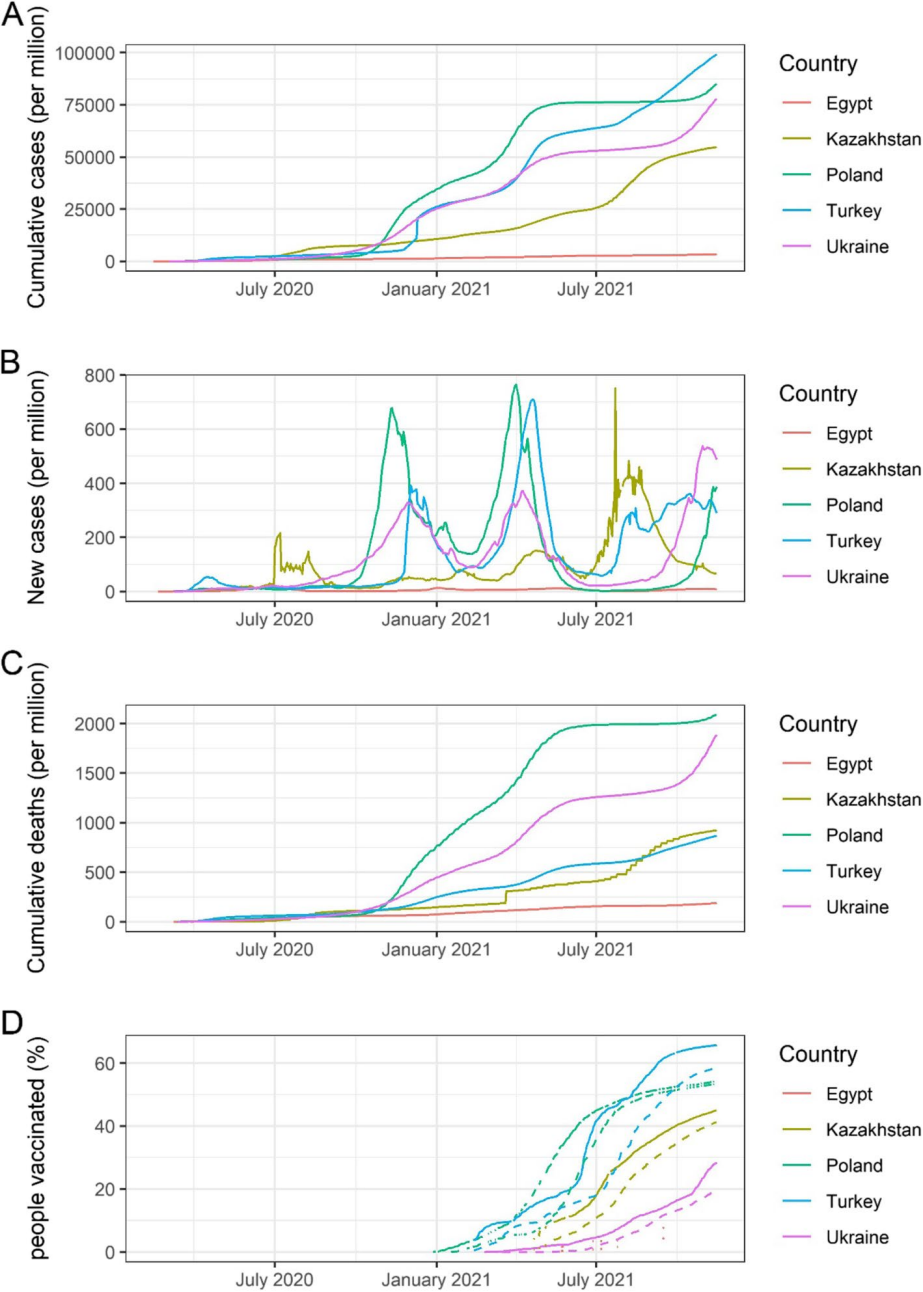
Cumulative COVID-19 cases, new daily cases, deaths, and vaccination coverage since the beginning of pandemic in Turkey, Egypt, Ukraine, Kazakhstan, and Poland. **A** Cumulative cases per million population in Turkey, Egypt, Ukraine, Kazakhstan, and Poland from 1 February 2020 to 15 November 2021, **B** New daily cases per million population in each country, **C** Cumulative deaths per million population in each country, **D** Percentage of people partially and fully vaccinated in each country. Solid line shows the proportion of total population partially vaccinated (received at least one dose). Dashed line shows the proportion of total population fully vaccinated. Vaccination data in Egypt was not available in majority of the time. Vaccination data in Poland was missing at several time periods. (Data source: Our World in Data [11] – accessed on 16 November 2021)

All five countries confirmed the first COVID-19 case in their respective countries between February and early March 2020. Most of the first cases were imported from Western Europe. Turkey, Ukraine and Poland have a relatively large number of cumulative cases (8.4 million, 3.2 milion, and 3.2 million), while Egypt and Kazakhstan have a smaller number of cumulative cases (0.3 million and 1.0 million) as of 16 November 2021 [10]. Cumulative cases, new cases, cumulative deaths, and vaccination coverage in Turkey, Egypt, Ukraine, Kazakhstan, and Poland from 1 February 2020 to 15 November 2021 are shown in Fig. 2. The number of cases, deaths, and vaccination coverage, as well as key health indicators by country, are summarised in Table 1.

**Table 1:** COVID-19 cases, deaths, and vaccine coverage, and baseline health indicators. The number of cases and deaths of COVID-19 and the number of cases and death per 1,000 population, vaccine doses, selected (pre-pandemic) health and economic indicators in Turkey, Egypt, Ukraine, Kazakhstan, and Poland are summarised. The number of cases and deaths of COVID-19, and total number of vaccine administered were collected from the WHO dashboard as of 16 November 2021 [10]. The number of people fully vaccinated and partially vaccinated were collected from the Our World In Data as of 15 November 2021 [11]

	**Turkey**	**Egypt**	**Ukraine**	**Kazakhstan**	**Poland**	**Global**
Population in 2020	84,339,067	102,334,404	43,733,762	18,776,707	37,846,611	7,845,261,000
% above 65 years	8.7%	5.3%	16.7%	7.7%	18.1%	9.09%
Cases	8,432,018	344,907	3,244,749	1,039,671	3,230,634	253,640,693
(per 1,000 population)	(99.98)	(3.37)	(74.19)	(55.37)	(85.36)	(32.33)
Deaths	73,746	19,567	77,985	17,549	79,161	5,104,899
(per 1,000 population)	(0.87)	(0.19)	(1.78)	(0.93)	(2.09)	(0.65)
Vaccine dose total	117,378,532	33,667,594	19,322,408	16,046,796	39,587,985	7,307,892,664
(per 100 population)	(139.17)	(32.9)	(44.18)	(85.46)	(104.6)	(93.15)
Vaccine dose-1 (million)	55.87	21.12	12.33	8.54	20.48	4,110
(%)	(66%)	(20%)	(28%)	(45%)	(54%)	(52%)
Vaccine dose-2 (million)	49.71	13.27	8.91	7.82	20.15	3,220
(%)	(58%)	(13%)	(20%)	(41%)	(53%)	(41%)
Vaccine type	Pfizer	Sinopharm, AstraZeneca	AstraZeneca, Pfizer, Sinopharm	Sputnik V	Pfizer, Moderna	
Vaccine roll out started	Apr 2, 2021	Jan, 2021	Feb 24, 2021	Feb 1, 2021	Dec 2, 2020	
	**Turkey**	**Egypt**	**Ukraine**	**Kazakhstan**	**Poland**	**EU average**
Life expectancy at birth in 2018 (years)	77	72	72	73	78	81
GDP per capita in 2019 (current US$)	9,127	3,019	3,659	9,812	15,693	34,913
Health expenditure in 2019 (% of GDP)	4.12	4.95	7.72	2.92	6.33	9.85
Hospital bed per 1,000 population in 2018	2.9	1.4	7.5	6.1	6.5	4.6
Physicians per 1,000 population in 2018	1.8	0.5	3.0	4.0	2.4	3.7

Trends in the observed numbers of cases similarly had three peaks among five countries, although the peaks of transmission were different (see Fig. 2B). The cumulative number of deaths in Poland and Ukraine (2.09 and 1.78 per thousand population) were higher than in the other three countries (see Fig. 2C) [10].

### Health sector preparedness and response

#### Health system and health finance

The five countries had developed different health systems and health insurance schemes before COVID-19, and health system reforms are ongoing.

Turkey has been implementing health reform initiatives since 2003 [18]. This programme improved governance, health financing, and health service delivery significantly, with heavy investment in health infrastructure [19]. The General Health Insurance Scheme (GHIS), funded by a tax surcharge on employers [20], covers 99% of all inhabitants, including over 3.6 million Syrian refugees. Health services are provided both by public and private sector facilities [19]. The GHIS ensures free treatment for various conditions, such as emergency care, occupational illness, childbirth, and infectious diseases [21]. Their health system transformation enabled the outbreak response to be effective and timely with relatively limited strain on the existing health system and capacity.

The Egyptian healthcare system is funded and managed by governmental, parastatal, and private sectors. The Health Insurance Organisation oversees basic health coverage for 60% of the population [22]. The Egyptian health system was revitalised in 2014 and improved the quality of care, health expenditure, availability, and accessibility of disease surveillance. According to the WHO’s assessment in 2020, Egypt has a solid capacity to respond to the outbreak [22].

Ukraine has the weakest health system in the post-Soviet Union countries [23]. In addition, six years of conflict in east Ukraine weakened it further. Public healthcare is still in transition from the highly centralised health system. Free healthcare is the principle; however, 58% of patients reported having made out-of-pocket payments in 2017 [24]. Unmet healthcare needs are a growing issue in Ukraine [25].

The health system in Kazakhstan is highly centralised, and public health service is dominant. One of the key challenges in healthcare reform is the considerable inequity in health financing per capita among the different geographical areas in the country. Another challenge is that 36% of the health expenditure comes from out-of-pocket payments [26]. Since 2017, all citizens are required to participate in employers’ contributions to the healthcare fund. This measure is expected to boost healthcare spending and generally improve services for patients [27].

The National Health Fund finances the healthcare system in Poland with the capitation payment system [28]. Citizens pay their health insurance through their employer, which is 9% deducted from personal income and covers core-family members. Healthcare is free for all citizens; in particular, the government is obliged to provide free healthcare to young children, pregnant women, people with disabilities, and the elderly [29]. A challenge in the healthcare system in Poland is that out-of-pocket expenditure accounts for more than 20% of health expenditure. The number of medical workers per 1,000 population is lower than the European Union (EU) average, while spending for prevention is less than half of the EU average.

**Fig. 3:**
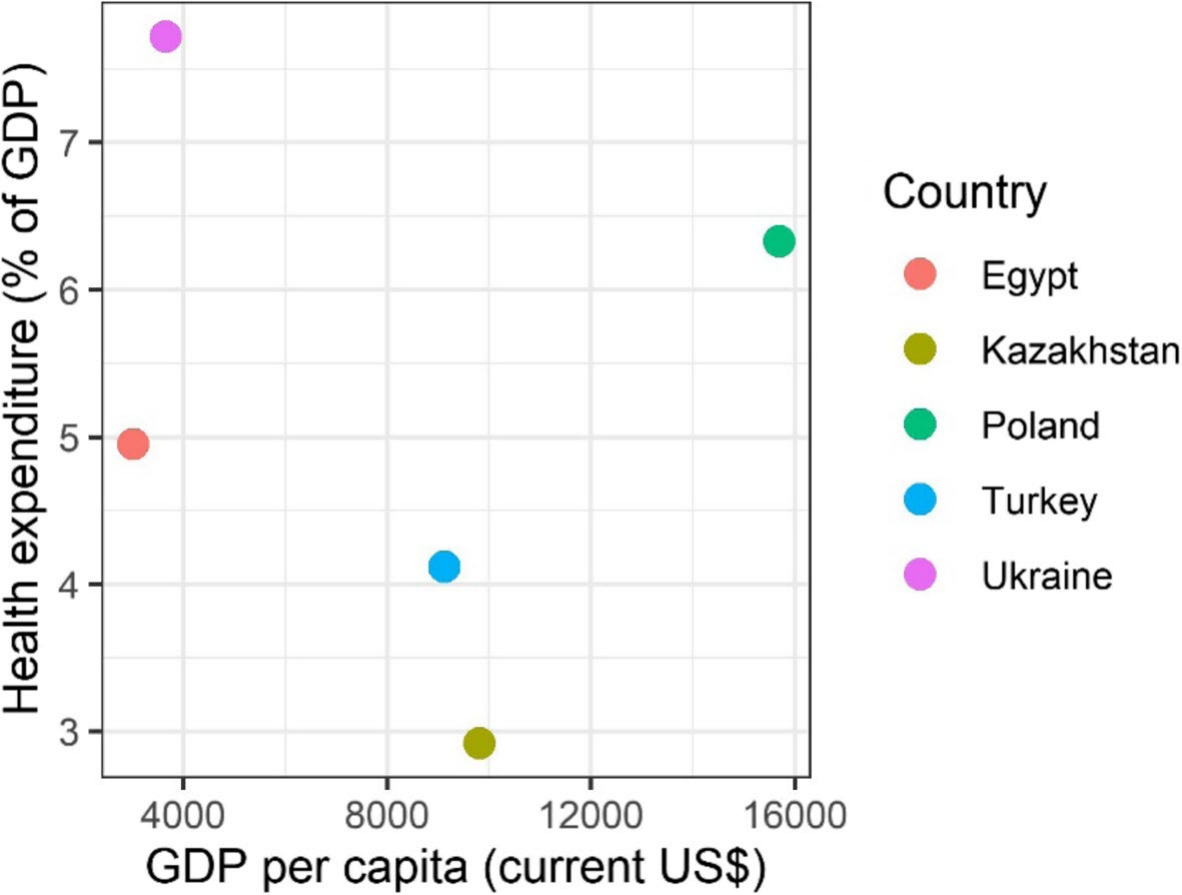
Health expenditure as percentage of GDP versus GDP per capita in 2019 for Turkey, Egypt, Ukraine, Kazakhstan, and Poland. The figure shows the relationship between GDP per capita (current US$) and health expenditure as percentage of GDP in Turkey, Egypt, Ukraine, Kazakhstan, and Poland. Among the five countries, health expenditure in terms of percentage of GDP is relatively lowest and highest in Kazakhstan and Ukraine respectively. (Data source: World Bank Database [16])

The healthcare expenditure (percentage of GDP) plotted over GDP per capita for the five countries show that the economy and health investment in each country varied (Fig. 3) [16]. Healthcare expenditures per GDP in Egypt, Turkey, and Kazakhstan was lower than 5%, which is below the recommended level of health financing. Although GDP in Poland was at the same level as for other EU countries, the health expenditure stayed low (6.2%), which may partly explain that the life expectancy in Poland is five years shorter than the EU average [30]. Ukraine has the highest health expenditure per GDP, and its health infrastructure and human resources are among the highest levels in Europe. However, Ukrainian medical care might not have met the standard of care in Europe, and their life expectancy is nine years shorter than the EU average (Table 1) [25].

#### National coordination of COVID-19 response activities

Turkey established an emergency operations centre immediately after the confirmation of COVID-19 in China and coordinated response activities through a Whole-of-Government approach. Turkey also established a scientific advisory board in the early stages [19, 31]. The Ukrainian government set up the Health Emergency Operation Committee in the MoH on 24 January and an inter-sectoral working group on 25 April 2020. Kazakhstan created an interdepartmental commission under the government to coordinate activities to prevent the spread of COVID-19 with all related ministries on 27 January 2020 [13].

#### COVID-19 testing capacity

Generally, probable cases and contacts with confirmed cases were tested by PCR in the five countries. The WHO has noted well-established COVID-19 surveillance systems in Turkey and Egypt [19, 22]. Case definitions of probable and confirmed cases were slightly different by country, though they follow WHO or the European Centre for Disease Control guidelines.

The five countries have made an effort to increase testing capacity during the pandemic. Turkish PCR testing capacity, one of the highest in the world, is supported by 453 laboratories, while Egypt established 40 laboratories [19, 22]. Ukraine had 96 test centres as of November 2020. PCR tests were conducted in nine laboratories at the oblast level and a national reference laboratory in Kazakhstan as part of the influenza surveillance programme. In Poland, 276 laboratories were carrying out testing at the end of January 2021. Total testing capacity exceeded 150,000 per day in Turkey, over 80,000 per day in Poland, and around 50,000 per day in Ukraine as of April 2021 [13].

Information about the implementation of contact tracing was largely absent except in Turkey. Turkey has more than 100,000 field teams conducting contact tracing [19, 22]. Potential contact persons were remotely monitored by audio or video call, if possible, in Kazakhstan [13].

#### Health infrastructure

The five countries rapidly increased the bed capacities to accommodate COVID-19 patients with the onset of the pandemic. Turkey has 563 hospitals dedicated to treating COVID-19 cases as of November 2020; up to 1,200 hospitals partly provided the care for COVID-19 cases. Over 25,000 ICU beds have already been available in Turkey. In addition, Turkey built two new pandemic field hospitals with a capacity of 1,000 beds [13]. Egypt has 750 COVID-19 designated hospitals with 35,152 beds, 2,218 ventilators, and 3,539 critical care beds. Ukraine increased the available beds for COVID-19 patients from 12,000 at the beginning of the pandemic to 53,445 in 582 designated hospitals as of 24 November 2020. In Kazakhstan, a mobile hospital in Nur-Sultan was assigned to deal with COVID-19 patients exclusively. Poland prepared at least one dedicated hospital in each province for case management [19, 22, 32, 33]. As of October 2020, approximately 8,000 beds and over 800 respirator beds were prepared in Poland [13].

The number of hospital beds in each country before the pandemic is summarised in Table 1. The number of tests, hospitals, and beds after the pandemic as of April 2021 is summarised in Table 2.

**Table 2:** COVID-19 pandemic response in Turkey, Egypt, Ukraine, Kazakhstan, and Poland. Coordination, test and treatment capacity, non-pharmaceutical interventions for COVID-19 response in Turkey, Egypt, Ukraine, Kazakhstan, and Poland were summarised. The border control, duration of school closure, and internal financial resource allocation for health care in five countries were also summarized. Ukraine received large financial aid from the United Nations, the World Bank

	**Turkey**	**Egypt**	**Ukraine**	**Kazakhstan**	**Poland**
National coordination	Yes	-	with WHO	Yes	-
	since Jan 2020-		since May 2020-	since Jan 2020-	
Existed legislation	Plan for pandemic influenza 2019	-	-	-	Infectious disease act 2008
COVID-19 dedicated facility and beds	563 (~ 1200) Hospitals	750 Hospitals	582 Hospitals	one mobile hospital	19 Hospitals
		35,152 beds		53, 445 beds	8,000 beds
PCR test capacity	453 labs	40 labs	-	-	-
	150,000 tests per day				
Lockdown measure	> 65 or < 20 yrs old	Night-time	full	full	full
	Mar 2020-	Mar-Jun, 2020	Mar-Jun, 2020	Mar-Jun, 2020	Mar 2020-
	Weekend & holiday	No-daytime restriction		Weekend	
	Apr 2020-			Jun 2020-	
Public entities / leisure places closure *	Yes Mar 2020-	Yes Mar-May, 2020	Yes Mar-May, 2020	Yes Mar-May, 2020	Yes Mar 2020-
Mask wearing in public place	Yes	Yes	Yes Apr 2020-	Yes Jul 2020 -	Yes Apr 2020-
Border closure	partial	complete	complete	complete	partial
		Mar-Jun, 2020			
	domestic travel ban	partial	domestic travel ban	domestic travel ban	
		Jun 2020-			
School closure	49 weeks	19 weeks	28 weeks	43 weeks	43 weeks
Finance for healthcare	-	-	UAH 1.25 billion	KZT 17 billion	PLN 7.5 billion
			US$ 45.2 million	US$ 3.9 million	US$ 2.0 billion

#### Healthcare workforce

Maintaining the healthcare workers for routine health services and COVID-19 responses was the largest challenge in the five countries. The strategies to keep the workforce in five countries were task shifting, financial incentives, and providing psychosocial care for them.

In Turkey, medical and dental residents were repurposed for the COVID-19 response. Poland mobilised non-specialised personnel, retired persons, medical students, and soldiers and assigned them certain tasks in line with their capacity. Ukraine reserved medical students to be hired as a surge capacity [13].

Turkey, Ukraine, and Poland increased the salary for those who work with COVID-19 patients by 100–300%. In Poland, the income loss was compensated for medical staff who were restricted to work out of their hospitals due to potential contacts with COVID-19 patients. Overtime payments and time off duty were ensured by law. Quarantined or isolated doctors received 100% of their salary in Poland and Ukraine. Turkey and Poland provided accommodation for healthcare workers who did not want to put their families at potential risk of infection [13].

In Ukraine, the MoH required healthcare personnel to pass WHO online courses on clinical management and infection prevention and control. WHO led training at 200 designated treatment hospitals and shared knowledge on COVID-19 treatment measures via video conferencing [13].

#### Medical supply

Due to the shutdown of factories in China, supply chains were considerably disrupted [34]. Many essential medical drugs were produced in China. Shortages of masks, gloves, and personal protective equipment (PPE) were reported worldwide [35]. Turkey, Egypt, Ukraine, and Poland reported a shortage of PPE in the early stages of the outbreak [19, 22, 36]. Turkey had strategized for the production and stockpile of drugs and PPE at a national level. Ukraine has received more than 65,000 items of PPE from WHO [37]. Poland has joined the EU’s medical equipment procurement mechanism for the purchase of gloves, goggles, face protectors, surgical masks, and clothing [38].

#### Physical distancing and non-pharmaceutical interventions

The five countries imposed regional or national quarantines, “lockdown” measures, between March and May 2020, and gradually lifted them in June 2020 or later. Business offices, restaurants, retail shops, and entertainment venues were closed. Public entities, parks, and beaches were closed. Mass gatherings and religious worship were generally prohibited [19, 22, 23, 33, 39]. Egypt has banned the two largest religious events in the country [22].

In Turkey, curfews have been imposed on those who have chronic illnesses or are aged either over 65 or under 20 years [19]. In Egypt, a night-time curfew was put in place, but no day-time lockdown was imposed [22]. The “partial lockdown” was later questioned because the lockdown period was prolonged without adequate suppression of disease transmission.

Ukraine, Kazakhstan, and Poland took strict restriction policies for all citizens. Ukraine and Poland divided countries into red, yellow, or green zones according to their local epidemic status [13]. Ukraine and Kazakhstan prohibited domestic travel from crossing regional borders as well as international travel [23, 33, 39]. This measure is called an “interstate lockdown,” which restricted the movement of people in a larger area than at household or individual level.

International travel was prohibited partially or fully in the five countries. Negative PCR results were required before entry, and travellers were quarantined at the border.

#### Health communication

Clear and transparent communication with the public is an important part of the pandemic response and for avoiding panic and misinformation, which may impinge on effective response activities. The official websites, online streaming, and social media became the main communication channels during the COVID-19 pandemic.

The MoH in Turkey established a public website and updated the number of cases and other information, e.g., guidelines, posters, and Q&A (questions and answers). Turkey used social media, including Twitter, Facebook, and Instagram accounts, to share information with the public. In Ukraine, an official recommendation of hand hygiene and respiratory etiquette was posted on several social media channels and the MoH website. Regular short daily briefings about the COVID-19 response were arranged and streamed online on the MoH website and television. Weekly briefings about the COVID-19 situation were distributed by text message or video. In Kazakhstan, visual posters were put at borders or transportation stations, and loudspeakers and mass media were used to disseminate COVID-19 prevention measures to the public regularly. In Poland, information was transmitted by website, Twitter, and Facebook through the official account of MoH or the Primary Health Office. A chatbot on the WhatsApp application also provided information about COVID-19.

Digital communication played a primary role in mass communication during the pandemic in Turkey, Ukraine, and Poland. Their investment in digital health had started prior to the pandemic.

### Impact on non-COVID-19 health services

Healthcare access to non-COVID-19 services, including essential health services, was reduced by both demand-side and supply-side constraints. In Ukraine, 14% of households could not access healthcare during the pandemic due to busy hospitals, shortage of medication, suspension of regular services, and lack of transportation [40]. In Poland, despite the significant growth of telemedicine, the total volume of services provided at primary care centres between March and November 2020 decreased by 9.6% compared with the same period of 2019 [33]. Home visits by midwives were minimised, and school nurses had no duties as schools were closed [33].

Telemedicine was promoted in Turkey, Ukraine, Kazakhstan, and Poland to maintain essential health services [13]. Ukraine, Kazakhstan, and Poland continued to provide routine medical assistance to pregnant women and children, patients receiving cancer treatment, as well as other life-threatening diseases while suspending routine screening or examination.

A hotline was created in Turkey, Ukraine, Kazakhstan, and Poland for COVID-19-related consultation or screening. These four countries provided free healthcare services related to COVID-19, including testing, treatment, and vaccination [13].

Turkey, Ukraine, and Poland reduced the number of admissions to the hospital, especially for elective surgery, though they continued to offer emergency surgery. Poland tentatively stopped routine childhood vaccination, though it resumed in April 2020. Ukraine observed a significant declining trend of routine vaccination in March–April 2020, but performance improved by July 2020 [13].

In Poland, training for resident doctors was stopped at the hospitals dedicated to COVID-19 patients. Some doctors in those hospitals have left their jobs as they could not continue their specialized practice, despite their salary being increased by the governmental compensation. There is a concern that the function of these hospitals might not be maintained even after the COVID-19 pandemic [13].

### Impact on the economy

COVID-19 is the biggest challenge that the global economy has experienced in the post-Second World War era. Because of the lockdown measures taken, domestic consumption declined by 40% in Kazakhstan [41]. Except for Turkey, the annual GDP growth rate declined in 2020 in comparison to the previous year for Egypt, Ukraine, Kazakhstan, and Poland (Table 3) [40–44]. Despite the well-diversified economy with advanced digitalisation, Poland experienced the first output contraction for over 20 years [44].

**Table 3:** Economic and social impact of COVID-19 pandemic in Turkey, Egypt, Ukraine, Kazakhstan, and Poland. The table summarised the most impacted industry, the poverty rate and the number of people who are under the poverty level, unemployment rate, and GDP growth before and after the COVID-19 pandemic (2019 and 2020) in Turkey, Egypt, Ukraine, Kazakhstan, and Poland. The pre-pandemic poverty rates were those of the average of 2018 and 2019 in each country. The table also shows the total funds for the economic stimulus policy

	**Turkey**	**Egypt**	**Ukraine**	**Kazakhstan**	**Poland**
Impacted sector	Service sector ^a^	Tourism	Trade	Oil and gas	Agriculture
		Cotton	Remittance	Service sector ^a^	Steel industry
		Suez Canal (trade)			Service sector ^a^
		Oil and gas			
poverty rate (%) in 2018/9 (< 5.5$ per day)	10.5%	4.1%	14.4%	6.0%	1.2%
	-	4.2 mil	6.3 mil	1.1 mil	-
poverty rate (%) in 2020 (< 5.5$ per day)	+ 2.1% 1.6 mil increase	4.3% 4.4 mil	20.6% 9.0 mil	12–14% 2.2–2.6 mil	-
Unemployment rate in 2019 (15–65 years)	-	-	8.1%	-	5.5%
Unemployment rate in 2020 (15–65 years)	-	9.6%	9.5%	-	6.5%
GDP growth in 2019 (annual %)	0.9%	3.6%	3.2%	4.5%	4.5%
GDP growth in 2020 (annual %)	1.8%	2.0%	-8.2%	-2.8% (Jan- Sep)	-2.7%
Total amount of fund for stimulus package ($US equivalent) % of GDP equivalent	100 bil TL	100 bil LE	-		-
	(15 bil $US)	(6.4 bil $US)		(10 bil $US)	
	13% of GDP	1.7% of GDP		5.7% of GDP	

^a^Service sector includes hospitality, retail, travel, and leisure industries

Unemployment increased in Egypt, Ukraine, and Poland [40, 44, 45]. The number of people living below the poverty line (US$ 5.50 per day for middle-income countries) increased in Turkey by 1.6 million, in Egypt by 0.2 million, in Ukraine by 2.7 million and in Kazakhstan by 1.1–1.5 million (Table 3) [40, 42–44].

Emergency funds were established to support domestic enterprises in the five countries to mitigate the economic fallout. Countries took similar measures, such as [41–44, 46, 47]: (i) affordable bank loans at discounted interest rates for businesses, (ii) financial support/cash transfers to poor households and affected individuals, (iii) support for firms’ payments such as short-term working capital or unpaid leave or subsidised salaries, and (iv) exemption from tax or social contributions, tax deferrals and subsidised loans for firms or targeted sectors.

These government policies have supported the economy to stay afloat, while Turkey and Egypt faced high inflation [42, 43]. Kazakhstan’s inflation was first driven by increased food prices, but later, the weak external demand, low oil prices, and subsequent exchange rate depreciation led to higher inflation [41]. The impact on the economy and its mitigation measures in each country are summarised in Table 3.

### Impact on education, gender, and civil liberties

The COVID-19 pandemic has negatively affected education for children. Schools were closed completely in the five countries for between 19 and 49 weeks as of 16 November 2021 [3]. E-learning or remote learning, such as video-based instruction, matching the skills of the teaching force to the new range of tasks and activities, could enhance the performance of schools. However, distance learning was challenging due to limited access to digital technologies in the five countries. The refugees and migrants in Turkey and Ukraine and general students in Kazakhstan have reported significant problems with the infrastructure of the internet [48–51]. Therefore, modified schooling and a better social security system were also warranted.

In Turkey, women have been more likely to lose their jobs and carry out domestic labour besides working remotely during the pandemic [4]. Uneven division of household labour by gender has continued or even aggravated. In Ukraine, women are disproportionately affected by the disease because women account for 82% of all health and social workers compared with the 70% worldwide average [40]. The pandemic and lockdowns have also led to an increase in domestic violence by 30% in Ukraine [40].

The shortage of PPE imposed a high risk of infection on healthcare workers. Medical professionals who pointed out the shortage of PPE and training for themselves were arrested in Egypt. Over 70 people, including health workers, journalists, and lawyers, were detained in Egypt between March and June 2020 [52]. One-sixth of the COVID-19 infections occurred in medical professionals in Poland as of April 2020. The Ministry of Health in Poland tried to prevent medical personnel from commenting on the pandemic regarding the shortage of PPE [36]. Censorship of speech in Egypt and Poland has highlighted the importance of balancing public health measures and civil liberties [36, 52].

### COVID-19 vaccination

Various types of COVID-19 vaccines have been rolled out globally, including 23 vaccines in different countries, of which eight have been approved for use by WHO (as of 15 November 2021) [53, 54].Turkey and Poland primarily used the vaccine made by Pfizer in the United States of America, Egypt used the Sinopharm vaccine from China, Ukraine used the Astra Zeneca vaccine made in India, and Kazakhstan used the Sputnik V vaccine from Russia. In Turkey, Poland, and Kazakhstan, 66%, 54%, and 45% respectively of the total population have received at least the first dose as of 15 November 2021. On the other hand, in Egypt and Ukraine, only 20% and 28% of the population have completed the first dose as of 15 November 2021 (Table 1, Fig. 2D).

Vaccine hesitancy in communities poses serious challenges in achieving adequate coverage. Ukraine and Egypt reported high vaccine hesitancy in both the general population and among healthcare professionals [55, 56]. The underlying causes of vaccine hesitancy were reported to be the lack of trust in the government-led healthcare sector in Ukraine [55]. Egyptian medical students mentioned that a lack of information about the adverse effects of the vaccine was the primary reason for vaccine hesitancy [56].

## Discussion

The COVID-19 pandemic has exposed weaknesses in healthcare systems in the emerging economies of Turkey, Egypt, Ukraine, Kazakhstan, and Poland, and highlighted the intricate link between health and economy. Individual-level testing, isolation, and contact tracing are effective public health interventions in mitigating the health and economic impact of the COVID-19 pandemic in comparison to population-level measures of lockdowns. Investment in pandemic preparedness through cross-sectoral collaboration and innovation of digital technologies for health and non-health sectors are essential to minimise the health and broader socioeconomic impact of the pandemic and for more equitable and sustainable development beyond the pandemic.

Turkey, Egypt, Ukraine, Kazakhstan, and Poland have been making public health system reform efforts that aim for equitable access and better quality of healthcare. A rigorous health care system and financing were fundamental in maintaining essential care and effective disease surveillance during the pandemic. Since the onset of the COVID-19 pandemic, these five emerging countries expanded the COVID-19 testing and treatment capacities over time. However, the five countries faced a shortage of healthcare workforce and medical supplies. Task-shifting or financial and psycho-social support for healthcare workers were useful to maintain the healthcare workforce and surge capacity, while professional motivation was also important to retain healthcare workers. Coordination of production, stockpile, and distribution of medical supplies at the national and international levels was a strategy to overcome the shortage of supplies.

The five countries took various social distancing or large-scale quarantine measures of lockdowns while individual contact tracing was not reported even as a strategy. The lockdowns placed a huge burden on the economy and education system in the five countries and increased the gender inequality in Turkey and Ukraine. Economic stimulus policy was taken to reactivate the economy after lockdown, but it has induced high inflation in the aftermath. Adverse economic outcomes are likely to further impact the health and well-being of the vulnerable population. Considering that economic and social impacts of lockdown, individual-based testing, isolation, and contact tracing should have been considered in the early stages of the outbreak to mitigate its economic impact, as this was the primary and key successful intervention in COVID-19 prevention and control measure in South Korea [57].

Telemedicine is an effective and affordable option, particularly for non-emergency healthcare or mental health counselling where direct patient-provider interaction is unnecessary [48]. Although telemedicine can increase access to healthcare by reducing transmission, implementation largely depends on accreditation, payment systems, and insurance. Some doctors expressed concerns about safety, quality, privacy, and accountability [58, 59].

Nevertheless, digital technologies played an important role in maintaining routine healthcare and health communication. Online education supplemented the missed in-person school classes; however, the network and technical issues remained. The evolution of digital technology is rapid, and digitalisation is a key for future development. One such example is the emergence of high quality open access e-courses and learning repositories [60]. These are particularly important in maintaining and developing healthcare professional education and competencies during times when resources for education and training are constrained.

While misinformation will potentially damage the effort of combatting the pandemic and need to be addressed by national governments, it has been noted that individual human rights and civil liberties would be restricted during the pandemic in the public interest [61]. In Egypt and Poland, medical professionals’ speech to the public was censored by the government. WHO emphasized that the public health measures should be necessary, reasonable, and non-discriminatory, complying with national and international law [61].

Based on the experience from the COVID-19 response, development of pandemic preparedness plans at both national and international levels and allocation of adequate resources are required. Financial mechanisms and cross-sectoral collaboration are essential to prepare for the next pandemic.

The core components of preparedness in the health sector are health infrastructure, including ICUs and ventilators, health workforce, laboratory and testing capacity, and the medical supply chain. These core capacities need to be maintained sustainably. Shortages of medical equipment, expertise, and personnel in some countries make the entire world vulnerable to the outbreak [62]. Building a resilient health system in all countries is fundamental for global health security.

COVID-19 has shown an inseparable link between public health and economy, as well as their critical interface for sustainable development. The work toward the SDGs attained in previous decades has in many ways been set back by the COVID-19 pandemic. The recovery process will be a chance to reconstruct a more equitable and resilient society.

This time-constrained analysis is limited by the lack of information available in English and comparable data about healthcare systems, detailed response strategies, and activities taken in each country. We recommend future research and analysis to assess the link between health security and sustainable economic development.

While many economic segments were negatively impacted, the pandemic accelerated the adoption of digital technologies, such as telemedicine, which will have long-term benefits. Digital technologies also helped the economies to keep operating via online platforms, including e-commerce, outsourcing, cloud computing, and remote working. Digitalisation will be one of the target investment areas in the future.

## Conclusions

At a global level, data from DEVEX on the scale of the investment response for COVID-19 indicates that healthcare systems, education and communication are the fourth, seventh and eighteenth commonest areas for investment [63]. This confirms the relative importance attached to these areas for improvement and are broadly consistent with our findings. The COVID-19 pandemic has exposed weaknesses in healthcare systems, particularly in the emerging economies of Turkey, Egypt, Ukraine, Kazakhstan, and Poland during 2020–2021. It illustrates how socio-economic well-being is linked to health provision. The early response to the pandemic focusing on individual-based isolation measures is not only an effective public health intervention but also a pragmatic and potentially optimal solution for mitigating the pandemic impact on the economy, education, and gender inequality. Smart investments in public health, including digital health and linking health security with sustainable development, are key for economic gain, social stability, and more equitable and sustainable development.

## Data Availability

The datasets generated and analysed during the current study are available in the repository of WHO, the World Bank, UNESCO and Our World in Data, https://covid19.who.int/ https://datatopics.worldbank.org/universal-health-coverage/coronavirus/ https://data.worldbank.org/indicator/SH.MED.BEDS.ZS https://data.worldbank.org/indicator/SH.MED.PHYS.ZS https://data.worldbank.org/indicator/SH.XPD.CHEX.GD.ZS https://data.worldbank.org/ https://en.unesco.org/covid19/educationresponse#durationschoolclosures https://www.euro.who.int/en/countries https://openknowledge.worldbank.org/handle/10986/35273 https://ourworldindata.org/coronavirus

## Abbreviations

COVID-19: Coronavirus Disease
EBRD: European Bank for Reconstruction and Development
WHO: World Health Organization
UNICEF: United Nations Children’s Fund
UNFPA: United Nations Fund for Population
UNDP: United Nations Development Programme
UNHCR: United Nations High Commission for Refugees
UNESCO: United Nations Educational, Scientific and Cultural Organization
UN Women: United Nations Entity for Gender Equality and the Empowerment of Women
GDP: Gross Domestic Product
EU: European Union
MoH: Ministry of Health
GHIS: General Health Insurance Scheme
PPE: Personal Protective Equipment
